# Maternal Circulating Vitamin D Level, Targeted Supplementation, and Perinatal Outcomes in Twin Pregnancy

**DOI:** 10.3390/nu16142239

**Published:** 2024-07-12

**Authors:** Sofia Roero, Agata Ingala, Silvana Arduino, Miriam Folino Gallo, Arianna Arese, Isabella Ferrando, Carlotta Bossotti, Alberto Revelli

**Affiliations:** Twin Pregnancy Care Unit, Gynecology and Obstetrics 2U, A.O.U. Città della Salute e della Scienza, Sant’Anna Hospital, Department of Surgical Sciences, University of Turin, Via Ventimiglia 1, 10126 Turin, Italy

**Keywords:** twin pregnancy, vitamin D, perinatal outcome, hypertensive disorders of pregnancy

## Abstract

Background: Vitamin D deficiency is associated with several obstetric complications in singleton pregnancy. The aim of this study was to assess whether vitamin D levels affect the outcomes of twin pregnancy and if targeted supplementation can improve perinatal outcomes. Methods: The serum vitamin D levels of 143 women with twin pregnancies were measured during their first trimester. Those with insufficient (10–30 ng/mL; IL group) or severely deficient (<10 ng/mL, DL group) vitamin D levels were supplemented. In the third trimester, vitamin D levels were reassessed. Perinatal outcomes of the IL and DL groups were compared with those of patients with sufficient levels (>30 ng/mL, SL group) since the beginning of pregnancy. Results: Women in the IL and DL groups had a higher incidence of hypertensive disorders of pregnancy (HDP) compared to the SL group (24.8% and 27.8% vs. 12.5%, p = 0.045): OR = 1.58 for the IL group and 1.94 for the DL group compared to the SL group. In patients whose vitamin D levels were restored after supplementation, HDP incidence was lower than in patients who remained in the IL or DL groups (23.4% vs. 27.3%) but higher than those who were always in the SL group (12.5%). Conclusions: Insufficient or severely deficient levels of vitamin D in the first trimester are associated with an increased risk of HDP in twin pregnancy. The beneficial effect of targeted vitamin D supplementation in reducing HDP seems limited.

## 1. Introduction

Twin pregnancy is associated with an increased rate of several maternal complications, including hypertensive disorders of pregnancy (HDP), preterm birth (PB), gestational diabetes mellitus (GDM), and fetal complications like growth restriction (FGR), malformations, and intertwin birthweight discrepancy [1,2,3,4]. Women expecting twins are more likely to develop vitamin deficiency throughout gestation; therefore, they need specific nutritional advice in order to follow a correct diet and achieve sufficient intake of both macro- and micronutrients [5,6,7].

Vitamin D is a fat-soluble, steroid hormone that acts as a regulator not only of calcium and bone metabolism but also as a modulator of cell proliferation, differentiation, and apoptosis [8]. Moreover, vitamin D has been proven to affect and influence several organs, such as the immune, endocrine, cardiovascular, and neurological systems [9,10,11].

In singleton pregnancy, it has been reported that vitamin D deficiency is associated with a higher incidence of obstetrical complications such as HDP, GDM, PB, and FGR [12,13,14,15,16]. However, there is still a paucity of data about the possible association between circulating vitamin D levels and perinatal outcomes in twin pregnancies. Recently, a significant influence of vitamin D deficiency on the risk of both GDM and higher birth weight discordance between twins was reported [8,17]. Overall, however, the available studies are heterogeneous and involve a small number of women, and several aspects are still to be clarified on the matter.

The aim of the present study was to assess whether vitamin D status in the first trimester of twin pregnancy affects perinatal outcomes and whether targeted supplementation could improve the incidence of obstetric and neonatal complications.

## 2. Materials and Methods

This is a retrospective cohort study. The initial sample of the study comprised women with twin pregnancies followed up at the Twin Pregnancy Care Unit of Sant’Anna Women’s Health Hospital in Turin (Italy) between January 2020 and December 2023.

In the considered time period, a sample of 332 women with twin pregnancies were screened at our institution; among them, 189 were excluded for the following reasons: preexisting diabetes mellitus and/or chronic hypertension, abnormal absorption (i.e., gastric bypass or celiac disease), spontaneous miscarriage, selective fetal reduction or elective termination of pregnancy, fetal aneuploidy, referral to our center after 14 weeks of gestation, delivery at another hospital, and insufficient data availability.

After careful application of the exclusion criteria, 143 women with twin pregnancies were finally included in the study; during pregnancy, all women were followed up by a team made up of four obstetricians who applied the following follow-up protocol, specific to twin pregnancies: clinical management of these women was based on monthly visits, during which blood pressure was measured and lab exams (blood count, urine test and culture, and serology for the main prenatal infections) were prescribed; the women also underwent a cervico-vaginal swab to screen for genital tract infections. During the first visit to our center, all women received detailed counseling regarding the peculiarities of twin pregnancy, explaining the heightened risk of maternal, fetal, and neonatal complications, as well as the follow-up protocol; also, they were informed about the possibility and limits of screening tests for fetal aneuploidy and counseled regarding the option to undergo invasive diagnosis. As per our protocol for the management of twin pregnancies, all women received oral low-dose acetylsalicylic acid (ASA) (100 mg a day) with the aim of preventing HDP, started before 16 weeks of gestation. All women in our cohort also received dietary recommendations from our professional nutritionists in order to reach appropriate weight gain and achieve a balanced diet leading to the correct intake of macro- and micronutrients.

As regards ultrasound monitoring, chorionicity was diagnosed in the first trimester by applying standard ultrasonographic (US) criteria [2], such as the presence of a T or lambda sign; however, chorionicity was confirmed after birth via post-partum placental examination. Serial growth-assessing US examinations every 4 weeks starting from week 20 were offered to all women with uncomplicated dichorionic twin pregnancies, while women with monochorionic twin pregnancies received US examinations every 2 weeks from week 16. As recommended [2], the US assessment of monochorionic twin pregnancies included measurement of the amount of amniotic fluid and Doppler velocimetry of the umbilical and middle cerebral arteries. Obviously, in the case of anomalies, US scans were scheduled more frequently. As recommended, at 19–21 weeks, a US scan assessing for detailed anatomy was performed; in addition, because of their increased risk of heart defects, women with monochorionic twin pregnancies also underwent a fetal echocardiogram. As international guidelines recommend [4], in women with uncomplicated dichorionic twin pregnancies, elective birth was scheduled between 37 + 0 and 37 + 6 weeks, whereas delivery in women with uncomplicated monochorionic twin pregnancies was planned between 36 + 0 and 36 + 6 weeks.

Gestational hypertensive disorders were defined as the sum of women with gestational hypertension (blood pressure higher than 140 mmHg systolic or 90 mmHg diastolic after 20 weeks of pregnancy) and women presenting with pre-eclampsia, i.e., gestational hypertension associated with proteinuria ≥300 mg/day, with or without organ damage (renal, hepatic, neurological, or hematological). Blood pressure was assessed during clinical examinations at least once a month while the woman was sitting quietly in an upright position; it was measured with the use of a manual sphygmomanometer, with the arm cuff positioned at heart level.

Circulating vitamin D levels were determined in the first trimester. Blood samples were collected via venipuncture into Vacutainer tubes (6.0 mL volume) containing a serum clot activator; determination of vitamin D was then performed using chemiluminescence immunoassay (CLIA). Vitamin D serum levels were classified as sufficient (>30 ng/mL), insufficient (10–30 ng/mL, IL), or severely deficient (<10 ng/mL, DL). When an insufficient or severely deficient serum level was detected, 25.000 U.I./2.5 mL oral solution containing 0.625 mg of cholecalciferol/vitamin D3 was administered once a week for a month and then once every two weeks. In the third trimester, circulating vitamin D levels were re-assessed.

Because of anonymous data collection and the study’s retrospective nature, the study was deemed exempt from approval by the local institutional review board. It fully adheres to the World Medical Association Declaration of Helsinki (as revised in 2013) and complies with the ethical standards of national and institutional committees on human experimentation. Written informed consent for the use of personal information was obtained from every participant in written form at the time of the first visit.

### Statistics

SPSS Statistical analysis software by SPSS Statistics Inc. IBM Corp—Armonk, NY, USA—(Released 2021; IBM SPSS Version 29.1) was used to analyze the data.

We compared the perinatal outcomes of women with sufficient circulating vitamin D levels in the first trimester (S group) vs. those of women with insufficient levels (I group) or severely deficient levels in the first trimester (SD group). After targeted vitamin D exogenous supplementation (performed only in I and SD cases), we repeated the same comparison in the third trimester. Parametric variables were compared using the two-tailed Student’s *t*-test, while non-parametric variables were compared with the Mann–Whitney U test; categorical data were compared by χ^2^ or Fisher’s exact tests. Adjusted odd ratios (aORs) were calculated for categorical data using binomial logistic (multivariate) regression analysis to correct for confounding factors such as maternal age, ethnicity, and mode of conception. A *p*-value < 0.05 was considered statistically significant.

## 3. Results

A sample of 143 women with twin pregnancies were followed up at the Twin Pregnancy Care Unit of Sant’Anna Women’s Health Hospital in Turin (Italy) and included in the study. Their basal clinical characteristics as well as the overall incidence of pregnancy complications appear in Table 1. Most non-Caucasian women in our population were North African (mainly Moroccan or Egyptian).

Women with severe vitamin D deficiency (SD group) or insufficiency (I group) were significantly younger, less frequently nulliparous, and less frequently Caucasian; they conceived spontaneously more frequently, while women with sufficient vitamin D levels (S group) more frequently conceived using medically assisted reproduction (Table 2). No difference in maternal BMI and chorionicity was found between the three groups (Table 2).

Table 3 shows the perinatal outcomes of the women divided into three groups according to circulating vitamin D levels in the first trimester. Hypertensive disorders of pregnancy (HDP) were significantly more frequent in women with severely deficient (SD group) or insufficient levels (I group) of vitamin D than in those with sufficient levels (S group). Multivariate analysis, performed to control for confounding factors such as maternal age, BMI, ethnicity, and parity, confirmed a significant association between insufficient or severely deficient first-trimester circulating vitamin D levels and HDP, with aOR = 1.58 for I group vs. S group (95% confidence interval: 1.23–2.12), and aOR = 1.94 for SD group vs. S group (95% confidence interval 1.52–3.61) (Table 3).

In the third trimester, after vitamin D supplementation was given throughout pregnancy to both the I group and SD group, 5 women were still severely deficient (SD–II group; 3.5%), 50 had insufficient levels (I–III group; 35.0%), and 88 had sufficient levels (S–III group; 61.5%). Table 4 shows the perinatal outcomes according to circulating vitamin D level in the third trimester. No significant difference was found between the three groups.

Table 5 shows the comparison among patients who had sufficient circulating vitamin D levels since the beginning of their pregnancy (S group, n = 24) vs. those who had insufficient or severely deficient levels in the first trimester (I/SD group; n = 119) and received vitamin D supplementation. Among the latter, some patients returned to sufficient vitamin D levels in the third trimester (I/SD-to-S; n = 64), whereas others did not, remaining with insufficient or deficient levels (I/SD-always; n = 55). Interestingly enough, the incidence of HDP was higher in the I/SD-always group than in the I/SD-to-S group (27.3% and 23.4%, respectively), although both groups had a higher incidence than the S group (12.5%; *p* = 0.046) (Table 5).

## 4. Discussion

Vitamin D deficiency is rather frequent in the general population, and even more common in pregnant women, making this condition a public health issue.

In our sample of women pregnant with twins, the rate of severe vitamin D deficiency or insufficiency reached 83.2% (119/143), a prevalence comparable to that reported in singleton pregnancies [18,19,20] but higher than previously found in multiple pregnancies [8,17]. Indeed, vitamin D status is influenced by many factors including maternal age, BMI, and dietary habits; it can also change based on season, latitude, skin pigmentation, and skin coverage [21,22]. In our population, women with severe vitamin D deficiency or insufficiency were younger, less often nulliparous, and more frequently non-Caucasian; this finding is coherent with the fact that population characteristics (especially age and skin pigmentation) influence vitamin D status. It should also be considered that most non-Caucasian women in our population were North African, and these women often cover most of their skin for religious or cultural reasons, thus reducing sun exposure; this, together with darker skin pigmentation on average, could justify the high incidence of non-Caucasian women with severe vitamin D deficiency or insufficiency. Moreover, only half of the women with sufficient vitamin D levels in our sample had a spontaneously conceived pregnancy; indeed, women undergoing medically assisted reproduction (MAR) are often checked for vitamin D levels before treatment and, if necessary, pharmacologically supplemented, since an association between vitamin D deficiency and spontaneous miscarriage or reduced MAR success rate has been reported [23,24,25].

It must also be considered that cutoffs for normal serum vitamin D values may slightly vary among studies and international guidelines, affecting the proportion of subjects with sufficient levels; a concentration of circulating vitamin D < 30 ng/mL is regarded as lower than normal by most authors [26,27,28,29,30]. The most frequently reported cutoffs are the following: >30 ng/mL represents a sufficient level, between 20 and 30 ng/mL is regarded as insufficiency, and <20 ng/mL is regarded as deficiency [28]. In the present study, in order to enhance differences, we decided to consider a level <10 ng/mL as real, severe deficiency and to include patients with a level between 10 and 20 ng/mL in the insufficiency group. A recent study contributed to this choice: it found a significant difference in vitamin D concentration between singleton and multiple pregnancies, showing that, surprisingly, women carrying twins have higher serum vitamin D levels than those with singleton pregnancies [31]. The authors suggested that new normality ranges, specific to twin pregnancy, should be evaluated [31].

Overall, our data show that vitamin D insufficiency or severe deficiency in the first trimester of twin pregnancy is related to a higher incidence of gestational hypertensive disorders (HDP), similar to what has been previously reported for singleton pregnancy [12,16,22,32,33,34,35]. In particular, women presenting severe vitamin D deficiency or insufficiency in the first trimester had an about 2-fold and 1.5-fold increased incidence of HDP, respectively, compared to those who were vitamin D sufficient from the first trimester. Of note, these data were observed despite all women being given low-dose (100 mg a day) acetylsalicylic acid (ASA) since the beginning of pregnancy.

Targeted vitamin D supplementation decreased the proportion of insufficient and severely deficient women, which was lower in the third trimester; however, some deficient and insufficient women were still detected, as a possible consequence of low compliance with supplementation, decreased absorption, skin type, etc.; these women (IL/DL-always group) still had a significantly higher incidence of HDP in comparison with those with sufficient vitamin D levels since the beginning of pregnancy (S group). Interestingly enough, women with insufficient or severely deficient levels in the first trimester who became sufficient in the third trimester, after vitamin D supplementation (I/SD-to-S group), also had a higher rate of HDP in comparison with those who were always sufficient (S group).

Our findings suggest that circulating vitamin D levels in the first trimester, and possibly even in the periconceptional period, could be crucial in determining the subsequent risk of HDP. Indeed, several authors have reported that vitamin D is important, especially in the early stages of pregnancy, and could play a role in implantation and placentation [36]; to confirm this idea, it has been demonstrated that the placenta is one of the main sites of conversion of the inactive precursor 25(OH)-D3 into the active form of vitamin D (1,25(OH)_2_-vitamin D). The key enzyme for this metabolic reaction is 1a-hydroxylase (CYP27B1), which has been shown to have high activity both in maternal decidua and fetal trophoblast [37,38]. Recently, it was reported that vitamin D stimulates human extravillous trophoblast invasion in vitro [39], has a crucial role in regulating decidual immunity, promotes antibacterial functions of the innate immune system, suppresses adverse inflammatory responses, and controls local inflammation [40,41,42]. In women with pre-eclampsia, DNA hyper-methylation in some key placental genes involved in vitamin D signaling has also been reported, suggesting that reduced placental vitamin D transfer, limiting the substrate required for implantation and early placental development, could contribute to the onset of pre-eclampsia [34].

Our data suggest that vitamin D supplementation might be associated with a slight, non-significant reduction in the incidence of HDP in twin pregnancies, though it seems that vitamin D levels in the first trimester are more significantly associated with this outcome. To the best of our knowledge, this is the first study to report on targeted vitamin D supplementation in twin pregnancy; other studies, but not all [41], have found a positive effect of vitamin D supplementation in reducing the risk of HDP in singleton pregnancy, especially when initiated before 20 weeks of gestation [35,43,44,45,46]. Furthermore, our data do not confirm the presence of an association between vitamin D status and the risk of GDM, nor a positive effect of vitamin D supplementation in reducing the incidence of GDM. Other studies have found an association between vitamin D status and GDM in singleton pregnancies [15,16,22,47,48] and in twin pregnancies [17]. Vitamin D supplementation in the periconceptional period or during singleton pregnancy has been proposed by a few studies [49,50,51]. Overall, further studies are needed to better clarify the role of vitamin D administration in preventing obstetrical and perinatal complications in twin pregnancy.

Limitations of the present study include the lack of precise information regarding the season in which vitamin D was measured, although it is likely that our cases were homogeneously distributed throughout the whole year, and the fact that this is a retrospective study. Also, it would have been interesting to evaluate vitamin D status in newborn twins, since a direct connection with maternal vitamin D levels has been reported [8]. On the other hand, the strengths of this study are that the sample size is bigger than that of other previously published studies [8,17], as well as the consistency in pregnancy management, criteria, and mode of vitamin D supplementation.

## 5. Conclusions

In conclusion, the present study shows that vitamin D insufficiency or severe deficiency in the first trimester is significantly associated with a higher incidence of HDP in twin pregnancy; this finding may suggest considering a periconceptional measurement of circulating vitamin D level and, when needed, targeted supplementation in order to avoid vitamin D severe deficiency or insufficiency in early pregnancy. The beneficial effect of vitamin D-targeted supplementation during the second and third trimesters of pregnancy appears limited and not very effective in significantly reducing the incidence of HDP in twin pregnancies. Still, assessing circulating vitamin D levels in the first trimester of pregnancy and giving targeted supplementation may be worthwhile in order to partly reduce the incidence of HDP and monitor mothers with twin pregnancies with low vitamin D levels and increased risk more strictly, thus leading to a prompt diagnosis and treatment of this complication.

## Figures and Tables

**Table 1:** Maternal characteristics of the 143 women included in the study and the overall incidence of some pregnancy complications. Data are shown as mean ± SD or as number and percentage (in parenthesis). BMI = Body Mass Index; GDM = gestational diabetes mellitus; HDP = hypertensive disorders of pregnancy; FGR = fetal growth restriction.

Population Characteristic/ Pregnancy Outcome	Statistics
Maternal age (years)	34.1 ± 5.3
BMI (kg/m^2^)	23.5 ± 4.8
Nulliparity	73 (51.0%)
Spontaneously conceived pregnancy	114 (79.7%)
Caucasian ethnicity	120 (83.9%)
Dichorionicity	79 (55.2%)
GDM	35 (24.5%)
HDP	33 (23.1%)
Gestational age at birth (weeks)	34.9 ± 2.3
FGR	23 (16.1%)
Delivery by cesarean section	101 (70.6%)
Birth weight (g)	2256 ± 480
Birth weight discordance between twins (%)	8.3 ± 4.4

**Table 2:** Maternal characteristics of the women included in the study divided by circulating vitamin D level in the first trimester. Data are shown as the mean ± SD or as the number and percentage (in parenthesis); *p*-values for categorical data refer to the analysis of distribution in all three groups. * = S group vs. I group, ** = S group vs. SD group, *** = I group vs. SD group. BMI = Body Mass Index; ns = non-significant.

	Sufficient Level (S Group; *n* = 24)	Insufficient Level (I Group; *n* = 101)	Severely Deficient Level (SD Group; *n* = 18)	*p*-Value
Maternal age (years)	36.2 ± 4.6	34.2 ± 4.9	31.4 ± 5.6	** 0.022 *^,^*** ns
BMI (kg/m^2^)	22.4 ± 3.3	23.5 ± 5.0	25.8 ± 6.6	ns
Nulliparity	18 (75.0%)	51 (50.5%)	4 (22.2%)	0.016
Spontaneous pregnancy	13 (54.2%)	84 (83.2%)	17 (94.4%)	0.038
Caucasian ethnicity	24 (100.0%)	90 (89.1%)	6 (33.3%)	<0.001
Dichorionicity	14 (58.3%)	55 (54.5%)	10 (55.6%)	ns

**Table 3:** Comparison of perinatal outcomes according to circulating vitamin D level in the first trimester. Data are shown as the mean ± SD or as the number and percentage (in parenthesis); *p*-values for categorical data refer to the analysis of distribution in all three groups. GDM = gestational diabetes mellitus; HDP = hypertensive disorders of pregnancy; FGR = fetal growth restriction; ns = non-significant.

	Sufficient Level (S Group; *n* = 24)	Insufficient Level (I Group; *n* = 101)	Severely Deficient Level (SD Group; *n* = 18)	*p*-Value
GDM	6 (25.0%)	26 (25.7%)	3 (16.7%)	ns
HDP	3 (12.5%)	25 (24.8%)	5 (27.8%)	0.045
Gestational age at birth (weeks)	35.0 ± 1.6	34.7 ± 2.3	35.7 ± 1.1	ns
FGR	6 (25%)	14 (13.9%)	3 (16.7%)	ns
Delivery by cesarean section	16 (66.7%)	73 (72.3%)	12 (66.7%)	ns
Birth weight (g)	2207 ± 422	2201 ± 449	2308 ± 322	ns
Birth weight discordance (%)	12.6 ± 6.3	9.3 ± 4.6	10.5 ± 5.8	ns

**Table 4:** Comparison of perinatal outcomes according to circulating vitamin D level in the third trimester. Data are shown as the mean ± SD or as the number and percentage (in parenthesis); *p*-values for categorical data refer to the analysis of the distribution in all three groups. GDM = gestational diabetes mellitus; HDP = hypertensive disorders of pregnancy; IUGR = intrauterine growth restriction; ns = non-significant.

	Sufficient Level (S–III Group; *n* = 88)	Insufficient Level (I–III Group; *n* = 50)	Severely Deficient Level (SD–III Group; *n* = 5)	*p*-Value
GDM	21 (23.9%)	14 (28.0%)	0	ns
HDP	18 (20.5%)	15 (30.0%)	0	ns
Gestational age at birth (weeks)	35.4 ± 1.6	35.2 ± 1.1	36.0 ± 1.2	ns
FGR	13 (14.8%)	10 (20.0%)	0	ns
Delivery by cesarean section	62 (70.5%)	36 (72.0%)	3 (60.0%)	ns
Birth weight (g)	2339 ± 344	2286 ± 433	2490 ± 281	ns
Birth weight discordance (%)	9.7 ± 4.6	9.8 ± 5.7	12.2 ± 6.1	ns

**Table 5:** Comparison of perinatal outcomes according to circulating vitamin D level: S group = patients with sufficient levels since the first trimester; I/SD-to-S group = patients with insufficient or severely deficient levels in the first trimester who became sufficient in the third trimester after Vit D supplementation; I/SD-always group = patients with insufficient or severely deficient levels in the first trimester who did not become sufficient in the third trimester despite Vit D supplementation. GDM = gestational diabetes mellitus; HDP = hypertensive disorders of pregnancy; IUGR = intrauterine growth restriction; ns = non-significant. *p*-values for categorical data refer to the analysis of the distribution in all three groups.

	S Group (*n* = 24)	I/SD-to-S Group (*n* = 64)	I/SD-Always Group (*n* = 55)	*p*-Value
GDM	6 (25%)	15 (23.4%)	14 (25.5%)	ns
HDP	3 (12.5%)	15 (23.4%)	15 (27.3%)	0.046
Gestational age at birth (weeks)	35.0 ± 1.6	35.5 ± 1.5	35.3 ± 1.3	ns
FGR	16 (66.7%)	46 (71.9%)	39 (70.9%)	ns
Delivery by cesarean section	6 (25%)	7 (10.9%)	10 (18.2%)	ns
Birth weight (g)	2207 ± 422	2381 ± 423	2172 ± 358	ns
Birth weight discordance (%)	12.6 ± 6.3	9.3 ± 3.1	12.0 ± 3.8	ns

## Data Availability

The data presented in this study are available from the corresponding author upon request. The data are not publicly available due to privacy restrictions.
